# About the associations of vitamin D deficiency and biomarkers of systemic inflammatory response with all-cause and cause-specific mortality in a general population sample of almost 400,000 UK Biobank participants

**DOI:** 10.1007/s10654-023-01023-2

**Published:** 2023-06-21

**Authors:** Sha Sha, Tafirenyika Gwenzi, Li-Ju Chen, Hermann Brenner, Ben Schöttker

**Affiliations:** 1 Division of Clinical Epidemiology and Aging Research, German Cancer Research Center (DKFZ), Im Neuenheimer Feld 581, 69120 Heidelberg, Germany; 2 Faculty of Medicine, University of Heidelberg, 69115 Heidelberg, Germany; 3 Division of Preventive Oncology, German Cancer Research Center (DKFZ), National Center for Tumor Diseases (NCT), 69120 Heidelberg, Germany; 4 German Cancer Consortium (DKTK), German Cancer Research Center (DKFZ), Heidelberg, Germany

**Keywords:** Vitamin D, All-cause mortality, Cardiovascular mortality, Cancer mortality, Systemic inflammatory response

## Abstract

It is unknown whether the well-known association between vitamin D deficiency and mortality could be explained by the immune system modulating effects of vitamin D, which may protect from a systemic inflammatory response (SIR) to adverse health conditions. This study aims to investigate the interrelationships of vitamin D deficiency, biomarkers of SIR, and mortality. We used multivariate logistic regression with adjustment for 51 covariates to assess the associations of vitamin D deficiency with disadvantageous levels of nine biomarkers of SIR in the UK Biobank cohort. Furthermore, we tested with Cox regression and mediation analysis whether biomarkers of SIR and vitamin D deficiency were independently associated with mortality. We included 397,737 participants aged 37–73 years. Vitamin D deficiency was associated with disadvantageous levels of all blood cell count-based biomarkers, but not with C-reactive protein (CRP)-based biomarkers after adjustment for body weight. Vitamin D deficiency and all biomarkers of SIR were significantly associated with all-cause mortality and mortality from cancer, cardiovascular and respiratory disease. The strength of these associations was unaltered if vitamin D deficiency and biomarkers of SIR were put in the same model. This finding was further supported by the mediation analyses. This study showed that vitamin D deficiency is associated with disadvantageous levels of blood cell count-based but not CRP-based biomarkers of SIR. Vitamin D deficiency and systemic inflammation were independently and strongly associated with mortality. The potential of clinical interventions against both vitamin D deficiency and underlying causes of systemic inflammation should be explored.

**Supplementary Information:**

The online version contains supplementary material available at 10.1007/s10654-023-01023-2.

## Background

Inadequate vitamin D status has become an increasing concern worldwide [1, 2]. It has been well recognized that vitamin D actions go far beyond the regulation of bone metabolism and calcium homeostasis. Vitamin D and its metabolites are carried into the circulation via binding to vitamin D receptor (VDR) after being hydroxylated by the key enzyme 25-hydroxyl vitamin D3-1α-hydroxylase (CYP27B1) [3–10]. Vitamin D exerts effects on numerous extra-skeletal body functions, including immune system regulation, cardiovascular function, and a series of cellular effects such as anti-proliferation, pro-differentiation, pro-apoptosis, and anti-inflammation [3–10]. To date, there is accumulating evidence from observational studies and Mendelian randomizations that demonstrate significant associations of low vitamin D status with increased all-cause mortality, cancer mortality, cardiovascular and respiratory disease-related mortality [11–19]. Moreover, meta-analyses of randomized controlled trials (RCTs) provided further support for the efficacy of vitamin D supplementation in reducing all-cause mortality and cancer mortality [20–24].

It has been suggested that a sufficient vitamin D status (≥ 50 nmol/L) may protect from atherosclerosis and tumorigenesis through anti-inflammatory activities [24, 25]. This has led to an interest in whether vitamin D sufficiency could prevent a systemic inflammatory response (SIR) to adverse health conditions. In the scientific literature, a SIR is most frequently examined for cancer patients [24, 26], but it has also been observed in patients with diabetes mellitus, cardiovascular disease (CVD) [27–30], and patients who undergo any kind of surgeries or intensive care [31, 32]. The SIR is generally associated with poor prognosis [24, 26, 33–35]. This leads to the hypothesis of whether the association of low vitamin D status with mortality might be explained by the anti-inflammatory effects of vitamin D, which could attenuate a SIR to various diseases or treatments of these diseases [36].

The SIR is characterized by changes in blood cell counts and acute-phase proteins such as C-reactive protein (CRP) [37–39], which allows to broadly categorize the biomarkers of SIR in blood cell count and CRP-based markers. There are two modified versions of GPS with different cut-off values for CRP and serum albumin in the calculation, known as modified GPS (mGPS) and high-sensitive mGPS (HS_mGPS) [40, 41]. The blood cell count-based markers include the neutrophil-to-lymphocyte ratio (NLR), the platelet-to-lymphocyte ratio (PLR), the lymphocyte-to-monocyte ratio (LMR), the neutrophil-platelet score (NPS), the systemic immune-inflammation index (SII), and the prognostic nutritional index (PNI) [26, 42–47].

To date, observational studies from the general population have reported cross-sectional associations of vitamin D status with CRP, NLR, and PLR [48, 49]; and a Mendelian randomization analysis with data from the UK Biobank suggested that a low vitamin D status was causally related to increased CRP levels [50]. However, there are few studies on the associations of vitamin D status with other biomarkers of SIR [26].

The objectives of this study were to investigate the interrelationships of low vitamin D status with nine biomarkers of SIR (CRP, mGPS, HS_mGPS, NLR, PLR, LMR, SII, PNI, and NPS) and all-cause and cause-specific mortality in the large UK Biobank cohort study.

## Materials and methods

### Data source

The UK Biobank is a prospective cohort study, including approximately half a million United Kingdom (UK) population aged between 40 and 69 years at recruitment from 2006 to 2010 [51]. Large-scale biomedical information was collected from the 22 assessment centers across England, Scotland, and Wales through touchscreen questionnaires, verbal interviews, and a wide range of physical and medical assessments [51]. Biological specimens such as blood, urine, feces, and hair were collected at the initial assessment visit [52]. Data on health outcomes of all UK Biobank participants were gathered through linkages to health care records, including the UK National Health Service (NHS) data, primary care data, cancer screening data, and disease-specific registers [53].

### Study population

Of the 502,411 baseline participants of the UK biobank, we excluded 54,145 individuals whose serum 25-hydroxyvitamin D [25(OH)D] measurement was not available, and 50,529 individuals who did not have information on any biomarkers of SIR at baseline, leaving 397,737 participants included in this study.

### Vitamin D status

Vitamin D status was defined with the cut-offs of the US-American Institute of Medicine [54]: 25(OH)D levels < 30 nmol/L reflect vitamin D deficiency, 25(OH)D levels of 30 to < 50 nmol/L indicate vitamin D insufficiency and 25(OH)D levels ≥ 50 nmol/L indicate sufficient vitamin D status. 25(OH)D concentrations were determined using the Chemiluminescent Immunoassay, a direct competitive method on the DiaSorin Liaison XL (manufactured by Diasorin S.p.A), and externally validated by RIQAS Immunoassay Speciality I scheme with 100% good quality assurance [55, 56].

### Biomarkers of systemic inflammatory response

The serum CRP level (mg/L) was determined using immunoturbidimetric high-sensitivity analysis on a Beckman Coulter AU5800. The serum albumin level was measured by bromocresol green (BCG) analysis on the same apparatus [57, 58]. The Beckman Coulter LH750 Hematology Analyzer was used to measure peripheral blood samples taken within 24 h of the blood draw and 31 parameters including neutrophil, lymphocyte, monocyte, and platelet counts were obtained [59–63]. The equations to obtain the nine biomarkers of SIR used in this research project are shown in Table 1 [26, 42–47].

**Table 1:** Equations for biomarkers of systemic inflammatory markers

Biomarkers		Equation
CRP based	CRP	Measured value, mg/L
	mGPS	0: CRP ≤ 10 mg/L and albumin ≥ 35 g/L 1: CRP > 10 mg/L and albumin ≥ 35 g/L 2: CRP > 10 mg/L and albumin < 35 g/L
	HS_mGPS	0: CRP ≤ 3 mg/L and albumin ≥ 35 g/L 1: CRP > 3 mg/L and albumin ≥ 35 g/L 2: CRP > 3 mg/L and albumin < 35 g/L
Blood cell count based	NLR	Neutrophil count/lymphocyte count
	PLR	Platelet count/neutrophil count
	LMR	Lymphocyte count/monocyte count
	SII	Platelet count × neutrophil count/lymphocyte count
	PNI	Serum albumin (g/L) + 0.005 × 1000 × lymphocyte count (10^9^/L)
	NPS	0: Neutrophils ≤ 7.5 × 10^9^/L and platelets ≤ 400 × 10^9^/L 1: Neutrophils > 7.5 × 10^9^/L or platelets > 400 × 10^9^/L 2: Neutrophils > 7.5 × 10^9^/L and platelets > 400 × 10^9^/L

CRP, C-reactive protein; HS_mGPS, High-sensitive mGPS; LMR, lymphocyte-to-monocyte ratio; mGPS, modified Glasgow prognostic score; NLR, neutrophil-to-lymphocyte ratio; NPS, neutrophil-platelet score; PLR, platelet-to-lymphocyte ratio; PNI, prognostic nutritional index; SII, systemic immune-inflammation index

### Mortality

Information regarding the dates and causes of death was obtained from the NHS for the duration between the enrolment and 12 November, 2021. We used the *10th revision of the International Statistical Classification of Diseases (ICD-10)* to identify causes of death, i.e., mortality due to CVD (I00-I99), cancer (C00-C97), and respiratory disease (J00-J99).

### Covariates

This study developed models based on the 49 baseline characteristics identified as statistically significant and independently associated with vitamin D deficiency in a previous analysis of the UK Biobank data (see Supplemental (Suppl.) Table 1) [18]. The methods of the assessment of these covariates were described previously [18]. We included 47 out of these 49 covariates because we excluded vitamin D/multivitamin use and CRP (which were highly related to our main variables of interest). In the end, we used 51 covariates because we added a history of cancer (except non-melanoma skin cancer), inflammatory bowel disease, periodontitis, and pulmonary embolism due to their importance in SIR research and mortality outcomes.

### Statistical analyses

#### General remarks

All statistical analyses were performed using SAS statistical software (version 9.4, SAS Institute, Inc., Cary, NC, USA). Schoenfeld residuals were used to test the proportional hazards assumption and no violations of this assumption were observed. We used multiple imputation with five imputed datasets to fill in missing values except for exposures and outcomes [64]. With few exceptions, most of covariates had missing values of less than 5% and none had more than 19.1% missing values. The proportion of missing values for each variable used in the analyses can be calculated from the numbers shown in Suppl. Table 1. We used the Markov chain Monte Carlo (MCMC) technique, using a single chain and assuming multivariate normality for a dataset with arbitrary missing patterns [65]. Results from imputed datasets were analyzed using the SAS procedure PROC MIANALYZE.

#### Disadvantageous levels of biomarkers of systemic inflammatory response and their association with mortality

No established cut-off values for the dichotomization of the continuous biomarkers NLR, PLR, SII, LMR, and PNI are available in the literature. To obtain such cut-offs, we firstly drew restricted cubic spline curves (RCS) with age and sex-adjusted Cox proportional hazard regression models with 5 knots located at the 10th, 25th, 50th, 75th, and 90th percentiles with the SAS macro of Desquilbet and Mariotti [67]. To choose a cut-off to dichotomize each biomarker, we selected one of the 5 knots of the RCS curve at which the association with all-cause mortality had a turning point towards higher/lower hazard ratios (HR). Our definition of a turning point was that the new direction needed to manifest at this point and not start at it. Thus, the chosen cut-off was usually one knot after the knot at which the new direction started. The rationale for this definition of a turning point was to obtain strong effect estimates in the exposed group of the dichotomized biomarker variable. If a dose–response association was U-shaped, only a knot at the end of the biomarker distribution (low or high levels), which is known to be associated with mortality from the literature, was chosen. Although a cut-off of 3 mg/L in general population samples is available from the literature for high-sensitive CRP, out of reasons of consistency, we also applied the method above to find the best suitable cut-off for our dataset. An exception was only made for the PLR, which did not show the expected dose–response relationship with mortality (see results chapter). Due to low numbers of patients with 2 points in the mGPS, HS_mGPS, and NPS, patients with 1 or 2 points were merged into the category of disadvantageous levels to obtain dichotomized variables for these scores.

The obtained cut-offs were subsequently used in Cox proportional hazard regression models to assess HR and 95% confidence intervals (95% CI) for the associations of all nine biomarkers of SIR with all-cause, CVD, cancer, and respiratory disease mortality. The models were progressively adjusted for age, sex, BMI, waist circumference, and vitamin D status. This analysis was carried out for the total population and stratified by age (< 65/ ≥ 65 years) and sex.

#### Association of vitamin D status and biomarkers of systemic inflammatory response

The dichotomized biomarkers of SIR were used as dependent variables in logistic regression models to assess their association with vitamin D status (independent variable with three categories: deficiency, insufficiency, and sufficient vitamin D). To account for the high number of statistical tests in this analysis, the false discovery rate (FDR) was applied to determine statistical significance (FDR < 0.05). This analysis was also carried out for the total population and stratified by age (< 65/ ≥ 65 years) and sex.

Overall, 5 models were developed with increasing adjustments. Model 1 includes age, sex, skin color, the latitude of the study center, and the calendar month of the blood draw. Model 2 adds socio-economic factors, model 3 lifestyle factors, model 4 body weight measures, and model 5 diseases, symptoms, and aspects of the general health status (for details about all 51 covariates summed up under these labels, see Suppl. Table 1). Model 4 is the main model because the covariates in model 5 could be potential intermediates from a clinical perspective. Variation inflation factors (VIF) were used to test if there was multicollinearity across the 51 variables of model 5 [66]. The median VIF of all the covariates and their categories was 1.5 and it ranged from 1.0 to 7.2. Thus, no factor had a VIF > 10, which would raise concerns regarding multicollinearity [66].

#### Association of vitamin D status and mortality

With the main model 4, Cox proportional hazards regression was used to assess the associations of vitamin D status with all-cause, CVD, cancer, and respiratory disease mortality. To address whether these associations of vitamin D status with mortality are independent of biomarkers of SIR, we added them one by one as covariates to the model. In addition, the same analysis was conducted with the continuous serum 25(OH)D concentration variable among subjects with vitamin D deficiency because this is a highly clinically relevant subpopulation with an approximately linear inverse relationship between 25(OH)D levels and mortality outcomes [13, 50]. No subgroup analyses by age and sex were performed because it is known from previous analyses of the UK Biobank that the associations of vitamin D status and mortality do not differ much by age and sex [18].

#### Mediation analysis

With the assumption of causality, we quantified the proportion of the total effect of vitamin D deficiency and vitamin D insufficiency on the mortality outcomes, which is mediated through biomarkers of SIR. We used the SAS macro of L. Valeri and T. J. VanderWeele for causal mediation analysis with adaptions for time-to-event analyses [68–70]. The covariates of model 4 were used to adjust the Cox proportional hazards regression models of the mediation analyses.

## Results

### Description of the study population

Overall, 397,737 participants aged between 37 and 73 years (median, 58 years) were included in the study (Table 2). A little more than half of the participants were females (53.1%). The median serum 25(OH)D level was 46.8 nmol/L and the majority of participants had either vitamin D deficiency (21.1%) or vitamin D insufficiency (34.4%). Most study participants scored 0 points for the mGPS (95.8%), HS_mGPS (77.4%), and NPS (96.1%), and only very few scored 2 points (less than 0.2%). Suppl. Table 1 describes all baseline characteristics used in the most comprehensively adjusted model.

**Table 2:** Baseline characteristics of the study population (N = 397,737)

Variables	N_total_ (%) ^a^	Median (IQR)
Sex		
Male	186,755 (46.9)	NA
Female	210,982 (53.1)	NA
Age (years)	397,737 (100.0)	58 (50; 63)
BMI (kg/m^2^)	396,196 (100.0)	26.7 (24.1; 29.9)
Smoking		
Never	217,643 (54.8)	NA
Former	137,932 (34.8)	NA
Current	41,560 (10.4)	NA
Alcohol consumption^b^		
Abstainer	123,409 (31.0)	NA
Low	159,230 (40.0)	NA
Medium	67,419 (17.0)	NA
High	47,679 (12.0)	NA
Hypertension	107,411 (27.0)	NA
Diabetes	19,953 (5.1)	NA
CHD	18,739 (4.7)	NA
History of any cancer^c^	29,710 (7.5)	NA
25(OH)D levels (nmol/L)	397,737 (100.0)	46.8 (32.3; 62.4)
Vitamin D status^d^		
Vitamin D deficiency	83,929 (21.1)	NA
Vitamin D insufficiency	136,692 (34.4)	NA
Vitamin D sufficiency	177,116 (44.5)	NA
CRP based biomarkers of SIR		
CRP	397,737 (100.0)	1.3 (0.7; 2.8)
mGPS		
0	381,157 (95.8)	NA
1	16,496 (4.2)	NA
2	84 (< 0.1)	NA
HS_mGPS		
0	307,861 (77.4)	NA
1	89,728 (22.6)	NA
2	148 (< 0.1)	NA
Blood cell based biomarkers of SIR		
NLR	397,737 (100.0)	2.1 (1.7; 2.8)
PLR	397,737 (100.0)	132.3 (105.4; 166.5)
LMR	397,737 (100.0)	4.2 (3.2; 5.3)
SII	397,737 (100.0)	529.0 (392.2; 716.8)
PNI	397,737 (100.0)	54.7 (52.2; 57.4)
NPS		
0	382,192 (96.1)	NA
1	14,811 (3.7)	NA
2	734 (0.2)	NA

25(OH)D, 25-hydroxyvitamin D; BMI, body mass index; CHD, coronary heart disease; CRP, C-reactive protein; HS_mGPS, High-sensitive mGPS; IQR, interquartile range; LMR, lymphocyte-to-monocyte ratio; mGPS, modified Glasgow prognostic score; NA, not applicable; NLR, neutrophil-to-lymphocyte ratio; NPS, neutrophil-platelet score; PLR, platelet-to-lymphocyte ratio; PNI, prognostic nutritional index; SD, standard deviation; SII, systemic immune-inflammation index; SIR, systemic inflammatory response

^a^Data from one imputed dataset. Does not include missing data

^b^Alcohol consumption: Low: women 0–19.99 g of ethanol per day (g/d) or men 0–39.99 g/d; Medium: women 20–39.99 g/d or men 40–59.99 g/d; High: women ≥ 40 g/d or men ≥ 60 g/d

^c^Any cancer except non-melanoma skin cancer

^d^Vitamin D deficiency: 25(OH)D < 30 nmol/L; Vitamin D insufficiency: 25(OH)D 30–50 nmol/L; Vitamin D sufficiency: 25(OH)D > 50 nmol/L

### Disadvantageous levels of biomarkers of systemic inflammatory response and their association with mortality

**Fig. 1:**
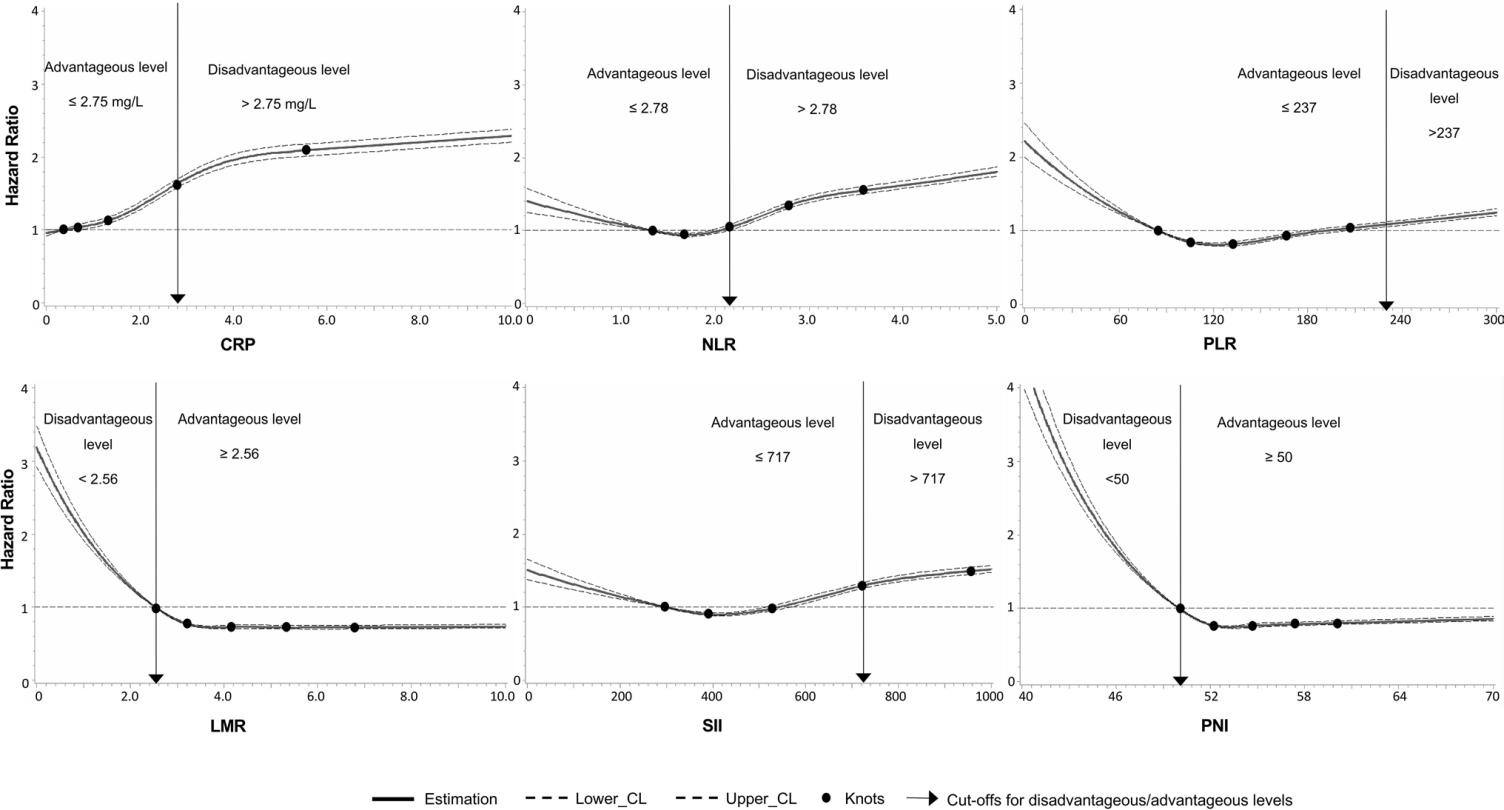
Age and sex adjusted dose–response relationships of biomarkers of systemic inflammatory response with all-cause mortality. CRP, C-reactive protein; LMR, lymphocyte to monocyte ratio; NLR, neutrophil to lymphocyte ratio; PLR, platelet to lymphocyte ratio; PNI, prognostic nutritional index; SII, systemic immune inflammation index. Restricted cubic splines with 5 knots, located at the 10th, 25th, 50th, 75th, and 90th percentiles of the biomarkers, were used to create the figure. These knots are represented by dots. The Y-axis represents the adjusted hazard ratio for all-cause mortality. The X-axis represents the measurement values of the respective biomarker. Horizontal green lines represent the hazard ratio of 1. Solid lines are estimates of hazard ratios and the dashed lines represent their 95% confidence intervals

During a maximum of 15 years of follow-up (median, 12.7 years), n = 29,548 study participants died. Figure 1 presents the age and sex-adjusted dose–response curves of the biomarkers of SIR with all-cause mortality. As cut-off values for the disadvantageous level, we chose the knot of the restricted cubic spline curve for each biomarker at which the association had a turning point towards higher/lower mortality. These were the knots at 2.75 mg/L (75th percentile) for CRP, 2.78 (75th percentile) for NLR, 237 (95th percentile) for PLR, 2.56 (10th percentile) for LMR, 717 (75th percentile) for SII, and 50 (10th percentile) for PNI. We considered levels above the cut-offs for CRP, NLR, PLR, and SII as disadvantageous, while levels below the cut-offs for LMR and PNI were also considered disadvantageous. This is because the latter two biomarkers were found to be inversely associated with mortality, as expected. An exception was made for the PLR, which in contrast to the previous studies showed higher mortality at low PLR levels than at high PLR levels [71]. Furthermore, there was no clear turning point at higher levels between 150 and 300, which were used as cut-off values in the previous literature [71]. Thus, to be comparable with previous studies, we chose the knot at the 95th percentile (PLR = 237).

We observed that disadvantageous levels of all biomarkers of SIR were strongly associated with increased all-cause mortality, CVD mortality, cancer mortality, and respiratory disease mortality in age and sex-adjusted models (Table 3). With further adjustment for body mass index (BMI) and waist circumference, the strength of the associations of CRP-based biomarkers of SIR with mortality was a little attenuated while this was not observed for the blood cell count-based biomarkers. After further adjustment for vitamin D status, the strength of the association between all biomarkers of SIR and mortality outcomes did not change to any relevant extent.

**Table 3:** Associations of dichotomized biomarkers of systemic inflammatory response with all-cause and cause-specific mortality

Mortality	Biomarkers of systemic inflammatory response, HR (95% CI), N = 397,737								
	CRP > 2.75 mg/L	mGPS ≥ 1	HS_mGPS ≥ 1	NLR > 2.78	PLR > 237	LMR < 2.56	SII > 717	PNI < 50	NPS ≥ 1
**All-cause mortality** (N_deaths_ = 29,548)									
Adjusted for age and sex	1.76 (1.72, 1.80)	2.18 (2.10, 2.27)	1.77 (1.73, 1.82)	1.48 (1.45, 1.52)	1.54 (1.48, 1.61)	1.54 (1.49, 1.58)	1.48 (1.45, 1.52)	1.52 (1.47, 1.56)	2.24 (2.14, 2.34)
Plus BMI and waist circumference	1.57 (1.54, 1.61)	1.91 (1.83, 1.99)	1.59 (1.55, 1.64)	1.47 (1.43, 1.51)	1.62 (1.55, 1.69)	1.52 (1.48, 1.57)	1.47 (1.43, 1.50)	1.51 (1.46, 1.55)	2.10 (2.01, 2.19)
Plus vitamin D status	1.56 (1.52, 1.60)	1.89 (1.81, 1.97)	1.58 (1.54, 1.62)	1.46 (1.42, 1.49)	1.61 (1.54, 1.68)	1.53 (1.48, 1.57)	1.45 (1.41, 1.48)	1.50 (1.46, 1.55)	2.04 (1.95, 2.13)
**CVD mortality** (N_deaths_ = 6,091)									
Adjusted for age and sex	2.00 (1.90, 2.11)	2.28 (2.09, 2.5)	2.04 (1.93, 2.15)	1.68 (1.60, 1.77)	1.37 (1.24, 1.52)	1.68 (1.58, 1.78)	1.64 (1.55, 1.72)	1.51 (1.41, 1.62)	2.56 (2.34, 2.80)
Plus BMI and waist circumference	1.61 (1.53, 1.70)	1.79 (1.64, 1.96)	1.64 (1.55, 1.73)	1.67 (1.58, 1.75)	1.51 (1.36, 1.67)	1.66 (1.56, 1.77)	1.61 (1.53, 1.70)	1.50 (1.40, 1.60)	2.32 (2.12, 2.54)
Plus vitamin D status	1.60 (1.51, 1.69)	1.77 (1.62, 1.94)	1.62 (1.54, 1.71)	1.65 (1.57, 1.74)	1.50 (1.35, 1.66)	1.66 (1.56, 1.77)	1.59 (1.51, 1.68)	1.50 (1.40, 1.60)	2.24 (2.05, 2.46)
**Cancer mortality** (N_deaths_ = 14,895)									
Adjusted for age and sex	1.62 (1.57, 1.67)	1.89 (1.78, 2.01)	1.62 (1.57, 1.68)	1.30 (1.26, 1.35)	1.51 (1.42, 1.61)	1.38 (1.32, 1.45)	1.34 (1.29, 1.39)	1.38 (1.32, 1.45)	1.77 (1.66, 1.9)
Plus BMI and waist circumference	1.52 (1.47, 1.58)	1.75 (1.65, 1.86)	1.53 (1.47, 1.58)	1.30 (1.26, 1.35)	1.56 (1.47, 1.66)	1.38 (1.32, 1.44)	1.33 (1.29, 1.38)	1.39 (1.32, 1.45)	1.70 (1.59, 1.82)
Plus vitamin D status	1.52 (1.46, 1.57)	1.74 (1.64, 1.85)	1.52 (1.46, 1.57)	1.29 (1.25, 1.34)	1.56 (1.46, 1.66)	1.38 (1.32, 1.44)	1.32 (1.28, 1.37)	1.38 (1.32, 1.45)	1.67 (1.57, 1.79)
**Respiratory mortality** (N_deaths_ = 2,086)									
Adjusted for age and sex	2.73 (2.51, 2.98)	3.73 (3.30, 4.22)	2.75 (2.52, 2.99)	2.02 (1.85, 2.20)	2.15 (1.87, 2.48)	2.03 (1.83, 2.25)	2.25 (2.06, 2.45)	1.82 (1.63, 2.03)	4.75 (4.21, 5.37)
Plus BMI and waist circumference	2.63 (2.40, 2.88)	3.25 (2.87, 3.67)	2.61 (2.39, 2.86)	1.96 (1.79, 2.14)	2.16 (1.87, 2.49)	1.97 (1.78, 2.19)	2.19 (2.01, 2.39)	1.76 (1.57, 1.96)	4.35 (3.85, 4.92)
Plus vitamin D status	2.59 (2.37, 2.84)	3.19 (2.82, 3.62)	2.56 (2.34, 2.80)	1.93 (1.77, 2.10)	2.13 (1.85, 2.46)	1.98 (1.78, 2.19)	2.14 (1.96, 2.33)	1.76 (1.58, 1.96)	4.13 (3.65, 4.67)

BMI, body mass index; CI, confidence interval; CRP, C-reactive protein; CVD, cardiovascular disease; HR, hazard ratio; HS_mGPS, High-sensitive mGPS; LMR, lymphocyte-to-monocyte ratio; mGPS, modified Glasgow prognostic score; NLR, neutrophil-to-lymphocyte ratio; NPS, neutrophil-platelet score; PLR, platelet-to-lymphocyte ratio; PNI, prognostic nutritional index; SII, systemic immune-inflammation index

Subgroup analyses by age and sex are presented in Suppl. Table 2 and 3, respectively. The associations of CRP-based biomarkers of SIR with all mortality outcomes were slightly stronger in younger age group (< 65 years) than in older age group (≥ 65 years). For blood count-based biomarkers, no consistent age difference was observed. Regarding sex differences, the CRP-based biomarkers of SIR showed stronger associations with all-cause, CVD and cancer mortality in males than in females, whereas the associations with respiratory disease mortality were comparable. The associations of blood cell count-based biomarkers of SIR with mortality outcomes were mostly comparable between the sexes for all mortality outcomes.

### Association of vitamin D status and biomarkers of systemic inflammatory response

Table 4 shows the cross-sectional associations of vitamin D deficiency and insufficiency (compared to sufficient vitamin D status) with disadvantageous levels of biomarkers of SIR in logistic regression models. In Model 1–3, which did not adjust for body weight, we observed that both vitamin D deficiency and insufficiency were consistently associated with the disadvantageous level of all CRP-based biomarkers of SIR. With adjustment for waist circumference and BMI in main Model 4, the odds ratios (ORs) were attenuated and close to the null effect value of 1. Adding waist circumference only led to almost the same results (data not shown). When additionally adjusted for diseases in Model 5, all OR were < 1.0, which could be a sign of overadjustment.

**Table 4:** Associations of vitamin D deficiency and insufficiency with disadvantageous levels of biomarkers of systemic inflammatory response in logistic regression models, N = 397,737

Biomarkers of systemic inflammatory response		Vitamin D Deficiency	Vitamin D Insufficiency	Vitamin D Sufficiency
		OR (95% CI), FDR	OR (95% CI), FDR	OR (95% CI), FDR
		*N*_*total*_ = 83,929	*N*_*total*_ = 136,692	*N*_*total*_ = 177,116
CRP based	CRP, N_case >2.75 mg/L_ (%)	25,271 (30.1)	35,106 (25.7)	38,982 (22.0)
	Model 1^a^	**1.65 (1.62, 1.69), < .001**	**1.27 (1.25, 1.29), < .001**	Ref
	Model 2^b^	**1.60 (1.56, 1.63), < .001**	**1.27 (1.24, 1.29), < .001**	Ref
	Model 3^c^	**1.37 (1.34, 1.40), < .001**	**1.18 (1.16, 1.20), < .001**	Ref
	Model 4^d^	1.01 (0.99, 1.04), 0.271	**0.97 (0.96, 0.99), 0.007**	Ref
	Model 5^e^	**0.96 (0.94, 0.99), 0.005**	**0.96 (0.94, 0.98), < .001**	Ref
	mGPS, N_case ≥1_ (%)	4659 (5.6)	5598 (4.1)	6323 (3.6)
	Model 1^a^	**1.65 (1.59, 1.73), < .001**	**1.17 (1.13, 1.22), < .001**	Ref
	Model 2^b^	**1.55 (1.48, 1.62), < .001**	**1.15 (1.11, 1.20), < .001**	Ref
	Model 3^c^	**1.28 (1.22, 1.34), < .001**	**1.06 (1.02, 1.10), 0.008**	Ref
	Model 4^d^	0.97 (0.93, 1.02), 0.233	**0.90 (0.87, 0.94), < .001**	Ref
	Model 5^e^	**0.92 (0.88, 0.97), 0.002**	**0.89 (0.86, 0.93), < .001**	Ref
	HS_mGPS, N_case ≥1_ (%)	23,179 (27.6)	31,694 (23.2)	35,003 (19.8)
	Model 1^a^	**1.68 (1.64, 1.71), < .001**	**1.27 (1.25, 1.29), < .001**	Ref
	Model 2^b^	**1.62 (1.58, 1.65), < .001**	**1.26 (1.24, 1.29), < .001**	Ref
	Model 3^c^	**1.38 (1.35, 1.42), < .001**	**1.18 (1.16, 1.20), < .001**	Ref
	Model 4^d^	1.03 (1.00, 1.05), 0.059	**0.97 (0.95, 0.99), 0.006**	Ref
	Model 5^e^	0.97 (0.95, 1.00), 0.050	**0.96 (0.94, 0.98), < .001**	Ref
Blood cell based	NPS, N_case ≥1_ (%)	4506 (5.4)	5187 (3.8)	5852 (3.3)
	Model 1^a^	**1.67 (1.60, 1.75), < .001**	**1.16 (1.11, 1.20), < .001**	Ref
	Model 2^b^	**1.54 (1.47, 1.61), < .001**	**1.13 (1.09, 1.17), < .001**	Ref
	Model 3^c^	**1.23 (1.17, 1.29), < .001**	1.04 (1.00, 1.08), 0.097	Ref
	Model 4^d^	**1.14 (1.09, 1.20), < .001**	0.99 (0.96, 1.04), 0.817	Ref
	Model 5^e^	**1.13 (1.07, 1.18), < .001**	1.01 (0.97, 1.06), 0.534	Ref
	NLR, N_case >2.78_ (%)	22,111 (26.3)	33,797 (24.7)	43,264 (24.4)
	Model 1^a^	**1.17 (1.15, 1.20), < .001**	**1.03 (1.01, 1.05), 0.001**	Ref
	Model 2^b^	**1.13 (1.11, 1.16), < .001**	**1.02 (1.00, 1.04), 0.030**	Ref
	Model 3^c^	**1.08 (1.06, 1.10), < .001**	1.00 (0.98, 1.01), 0.770	Ref
	Model 4^d^	**1.09 (1.07, 1.12), < .001**	1.01 (0.99, 1.03), 0.291	Ref
	Model 5^e^	**1.11 (1.08, 1.13), < .001**	**1.03 (1.01, 1.05), 0.003**	Ref
	PLR, N_case >237_ (%)	4418 (5.3)	6598 (4.8)	9009 (5.1)
	Model 1^a^	**1.07 (1.02, 1.11), 0.003**	**0.96 (0.93, 0.99), 0.013**	Ref
	Model 2^b^	1.04 (1.00, 1.08), 0.074	**0.95 (0.92, 0.98), 0.002**	Ref
	Model 3^c^	1.02 (0.98, 1.07), 0.406	**0.93 (0.90, 0.96), < .001**	Ref
	Model 4^d^	**1.13 (1.08, 1.18), < .001**	1.00 (0.96, 1.03), 0.880	Ref
	Model 5^e^	**1.17 (1.12, 1.22), < .001**	1.04 (1.00, 1.07), 0.060	Ref
	LMR, N_case <2.56_ (%)	8409 (10.0)	13,680 (10.0)	18,326 (10.4)
	Model 1^a^	**1.08 (1.04, 1.11), < .001**	1.00 (0.97, 1.02), 0.880	Ref
	Model 2^b^	**1.05 (1.02, 1.08), 0.003**	0.99 (0.97, 1.02), 0.500	Ref
	Model 3^c^	**1.04 (1.01, 1.07), 0.027**	0.98 (0.96, 1.01), 0.195	Ref
	Model 4^d^	**1.05 (1.02, 1.09), 0.003**	0.99 (0.97, 1.02), 0.664	Ref
	Model 5^e^	**1.06 (1.03, 1.10), 0.001**	1.01 (0.99, 1.04), 0.443	Ref
	SII, N_case >717 mg/L_ (%)	23,213 (27.7)	33,921 (24.8)	42,207 (23.8)
	Model 1^a^	**1.28 (1.26, 1.31), < .001**	**1.07 (1.05, 1.09), < .001**	Ref
	Model 2^b^	**1.25 (1.22, 1.27), < .001**	**1.06 (1.04, 1.08), < .001**	Ref
	Model 3^c^	**1.17 (1.15, 1.20), < .001**	**1.04 (1.02, 1.06), < .001**	Ref
	Model 4^d^	**1.17 (1.14, 1.20), < .001**	**1.04 (1.02, 1.06), < .001**	Ref
	Model 5^e^	**1.18 (1.15, 1.20), < .001**	**1.05 (1.04, 1.07), < .001**	Ref
	PNI, N_case <50_ (%)	8320 (9.9)	13,047 (9.5)	17,550 (9.9)
	Model 1^a^	**1.14 (1.10, 1.17), < .001**	1.01 (0.99, 1.04), 0.391	Ref
	Model 2^b^	**1.09 (1.06, 1.12), < .001**	0.99 (0.97, 1.02), 0.716	Ref
	Model 3^c^	**1.07 (1.04, 1.10), < .001**	0.98 (0.96, 1.01), 0.186	Ref
	Model 4^d^	**1.07 (1.04, 1.11), < .001**	0.99 (0.97, 1.02), 0.534	Ref
	Model 5^e^	**1.10 (1.06, 1.14), < .001**	1.02 (0.99, 1.04), 0.218	Ref

CI, confidence interval; CRP, C-reactive protein; HS_mGPS, High-sensitive mGPS; LMR, lymphocyte-to-monocyte ratio; mGPS, modified Glasgow prognostic score; NA, not applicable; NLR, neutrophil-to-lymphocyte ratio; NPS, neutrophil-platelet score; OR, odds ratio; PLR, platelet-to-lymphocyte ratio; PNI, prognostic nutritional index; SII, systemic immune-inflammation index

Numbers in bold indicate statistical significance of 0.05 level based on the nominal *p*-value

^a^Model 1 is adjusted for age, sex, skin colour, latitude of study center and calendar month of attending the assessment center

^b^Model 2 is adjusted for model 1 covariates plus socio-economic factors (education, Townsend deprivation index, no. of individuals in household, and household income)

^c^Model 3 is adjusted for model 2 covariates plus life-style factors (smoking, alcohol consumption, physical activity, frequency of visiting friends/family and consumption of oily fish, cereal, processed meat, milk, bread and spread), time spend outdoors in summer and winter, ease of skin tanning, use of sun screen/UV protection, and solarium/sunlamp use

^d^Model 4 is adjusted for model 3 covariates plus weight variables (body mass index and waist circumference)

^e^Model 5 is adjusted for model 4 covariates plus diseases & symptoms (diabetes, stroke, cancer, coronary heart disease, chronic obstructive pulmonary disease, history of pulmonary embolism, inflammatory bowel disease, periodontitis, arthritis, osteoporosis, gout, Parkinson, depressed mood, and tiredness/lethargy), biomarkers (estimated glomerular filtration rate, HbA_1c_, HDL cholesterol, systolic blood pressure, diastolic blood pressure, forced expiratory volume in 1-s, and hand grip strength), and general health status (no. of drugs, no of chronic diseases, disability, and general self-rated health)

This pattern was not observed for blood cell-based biomarkers of SIR. With the exception of NPS, increasing adjustment did not lead to strong attenuations in the associations with vitamin D deficiency, which remained statistically significantly associated with all blood cell count-based biomarkers of SIR in main Model 4 and the most comprehensively adjusted Model 5. With one exception of a weak, but statistically significant association of SII with vitamin D insufficiency, the latter was not associated with the blood cell-based biomarkers of SIR in main Model 4.

Subgroup analyses for age and sex were conducted only for the comparison of vitamin D deficiency and sufficiency with main Model 4. Regarding age, no large differences were observed between older (≥ 65 years) and younger (< 65 years) study participants but PLR, LMR, and PNI were only statistically significantly associated with vitamin D deficiency in the younger age group (Suppl. Table 4). Regarding sex, results for women were comparable to those in the total population (Suppl. Table 5). The same applied to most biomarkers of SIR among men. However, PLR and LMR were not statistically significantly associated with vitamin D deficiency among men. In contrast, a weak, but statistically significant association of vitamin D deficiency with HS_mGPS was detected among males (OR, 95% CI 1.05, 1.01; 1.09).

### Association of vitamin D status and mortality

We observed that people with vitamin D deficiency had 35%, 40%, 20%, and 66% statistically significantly increased all-cause mortality, CVD mortality, cancer mortality, and respiratory disease-related mortality, respectively, compared to people with sufficient vitamin D (Table 5). Furthermore, study participants with vitamin D insufficiency had statistically significant 9%, 12%, 5%, and 27% increased all-cause mortality, CVD, cancer, and respiratory mortality, respectively, compared to people with sufficient vitamin D. These effect estimates remained essentially unchanged when any biomarker of SIR was added to the model (the maximum HR difference was 0.03). The same pattern was observed when the continuous 25(OH)D level variable was used and the analysis was restricted to subjects with vitamin D deficiency (Table 6).

**Table 5:** Associations of vitamin D deficiency and insufficiency with mortality outcomes when biomarkers of systemic inflammatory response are added to the main multivariate Cox proportional hazards regression model, N = 397,737

Outcome: Mortality	Covariates adjusted for									
	Model 4 ^a^	Model 4 + CRP	Model 4 + mGPS	Model 4 + HS_mGPS	Model 4 + NLR	Model 4 + PLR	Model 4 + LMR	Model 4 + SII	Model 4 + PNI	Model 4 + NPS
**Exposure: Vitamin D deficiency (n = 83,929) versus sufficient vitamin D status (n = 177,116)**, **HR (95%CI)**										
All-cause (N_deaths_ = 19,545)	1.35 (1.30, 1.39)	1.35 (1.30, 1.39)	1.35 (1.30, 1.39)	1.34 (1.30, 1.39)	1.34 (1.29, 1.39)	1.34 (1.30, 1.39)	1.35 (1.30, 1.39)	1.33 (1.29, 1.38)	1.35 (1.30, 1.39)	1.34 (1.30, 1.39)
CVD (N_deaths_ = 4002)	1.40 (1.30, 1.51)	1.40 (1.30, 1.51)	1.41 (1.30, 1.51)	1.40 (1.30, 1.51)	1.39 (1.29, 1.50)	1.40 (1.30, 1.51)	1.40 (1.30, 1.51)	1.39 (1.29, 1.49)	1.40 (1.30, 1.51)	1.40 (1.30, 1.51)
Cancer (N_deaths_ = 9833)	1.20 (1.14, 1.26)	1.20 (1.14, 1.25)	1.20 (1.14, 1.26)	1.19 (1.14, 1.25)	1.19 (1.13, 1.25)	1.19 (1.14, 1.25)	1.20 (1.14, 1.25)	1.19 (1.13, 1.25)	1.19 (1.14, 1.25)	1.19 (1.14, 1.25)
Respiratory (N_deaths_ = 1374)	1.66 (1.47, 1.89)	1.66 (1.46, 1.88)	1.66 (1.47, 1.89)	1.65 (1.45, 1.87)	1.65 (1.45, 1.87)	1.66 (1.46, 1.88)	1.67 (1.47, 1.89)	1.63 (1.43, 1.85)	1.66 (1.47, 1.89)	1.65 (1.45, 1.87)
**Exposure: Vitamin D insufficiency (n = 136,692) versus sufficient vitamin D status (n = 177,116)**, **HR (95%CI)**										
All-cause (N_deaths_ = 21,812)	1.09 (1.06, 1.12)	1.09 (1.06, 1.13)	1.10 (1.07, 1.13)	1.09 (1.06, 1.13)	1.09 (1.06, 1.12)	1.09 (1.06, 1.13)	1.09 (1.06, 1.12)	1.09 (1.06, 1.12)	1.09 (1.06, 1.13)	1.09 (1.06, 1.13)
CVD (N_deaths_ = 4385)	1.12 (1.05, 1.19)	1.12 (1.05, 1.19)	1.12 (1.06, 1.20)	1.12 (1.05, 1.19)	1.12 (1.05, 1.19)	1.12 (1.05, 1.19)	1.12 (1.05, 1.19)	1.12 (1.05, 1.19)	1.12 (1.05, 1.19)	1.12 (1.06, 1.20)
Cancer (N_deaths_ = 11,414)	1.05 (1.01, 1.09)	1.05 (1.01, 1.09)	1.05 (1.01, 1.10)	1.05 (1.01, 1.09)	1.05 (1.01, 1.09)	1.05 (1.01, 1.09)	1.05 (1.01, 1.09)	1.05 (1.01, 1.09)	1.05 (1.01, 1.09)	1.05 (1.01, 1.09)
Respiratory (N_deaths_ = 1425)	1.27 (1.14, 1.41)	1.27 (1.14, 1.41)	1.27 (1.14, 1.42)	1.27 (1.14, 1.41)	1.27 (1.14, 1.41)	1.27 (1.14, 1.42)	1.27 (1.14, 1.41)	1.26 (1.13, 1.41)	1.27 (1.14, 1.42)	1.28 (1.14, 1.42)

CI, confidence interval; CRP, C-reactive protein; HR, hazard ratio; HS_mGPS, High-sensitive mGPS; LMR, lymphocyte-to-monocyte ratio; mGPS, modified Glasgow prognostic score; NA, not applicable; NLR, neutrophil-to-lymphocyte ratio; NPS, neutrophil-platelet score; PLR, platelet-to-lymphocyte ratio; PNI, prognostic nutritional index; SII, systemic immune-inflammation index

^a^The model is adjusted for covariates in Model 4 (see legend of Table 4)

**Table 6:** Hazard ratios for the association of 25(OH)D levels per 5 nmol/L with mortality outcomes among subjects with vitamin D deficiency with and without adjustment for biomarkers of systemic inflammatory response, N=83,929

HR (95% CI) per 5 nmol/L increase of 25(OH)D levels in subjects with vitamin D deficiency (25(OH)D < 30 nmol/L)										
	Covariates adjusted for									
Mortality	Model 4^a^	Model 4 + CRP	Model 4 + mGPS	Model 4 + HS_mGPS	Model 4 + NLR	Model 4 + PLR	Model 4 + LMR	Model 4 + SII	Model 4 + PNI	Model 4 + NPS
All-cause (N_deaths_ = 7736)	0.87 (0.85, 0.89)	0.87 (0.86, 0.89)	0.87 (0.85, 0.89)	0.87 (0.86, 0.89)	0.87 (0.86, 0.90)	0.88 (0.86, 0.90)	0.88 (0.86, 0.90)	0.88 (0.86, 0.90)	0.87 (0.85, 0.89)	0.87 (0.86, 0.90)
CVD (N_deaths_ = 1706)	0.86 (0.82, 0.90)	0.86 (0.82, 0.90)	0.86 (0.82, 0.90)	0.86 (0.82, 0.90)	0.86 (0.82, 0.90)	0.86 (0.82, 0.90)	0.86 (0.82, 0.90)	0.86 (0.82, 0.91)	0.86 (0.82, 0.90)	0.86 (0.82, 0.90)
Cancer (N_deaths_ = 3481)	0.91 (0.88, 0.94)	0.91 (0.88, 0.94)	0.91 (0.88, 0.94)	0.91 (0.88, 0.95)	0.91 (0.88, 0.94)	0.91 (0.88, 0.95)	0.91 (0.88, 0.94)	0.91 (0.88, 0.95)	0.91 (0.88, 0.94)	0.91 (0.88, 0.94)
Respiratory (N_deaths_ = 661)	0.79 (0.73, 0.86)	0.79 (0.74, 0.86)	0.79 (0.73, 0.86)	0.79 (0.73, 0.86)	0.80 (0.74, 0.86)	0.80 (0.74, 0.86)	0.80 (0.74, 0.86)	0.80 (0.74, 0.87)	0.79 (0.73, 0.86)	0.80 (0.74, 0.86)

25(OH)D, 25-hydroxyvitamin D; CI, confidence interval; CRP, C-reactive protein; HR, hazard ratio; HS_mGPS, High-sensitive mGPS; LMR, lymphocyte-to-monocyte ratio; mGPS, modified Glasgow prognostic score; NA, not applicable; NLR, neutrophil-to-lymphocyte ratio; NPS, neutrophil-platelet score; PLR, platelet-to-lymphocyte ratio; PNI, prognostic nutritional index; SII, systemic immune-inflammation index

^a^The model is adjusted for covariates in Model 4 (see legend of Table 4)

### Mediation analysis

Suppl. Tables 6 and 7 present the results of the mediation analyses for vitamin D deficiency and vitamin D insufficiency, respectively. The total effects estimated for the association of vitamin D deficiency and insufficiency with the mortality outcomes were consistent with the findings shown in Table 5. The proportion mediated of the total effect of vitamin D deficiency on all-cause mortality ranged between - 0.3 and 3.7% for the nine biomarkers of SIR, with a median of 1.1%. The median and range of the proportion mediated were similar for CVD mortality (median, 1.0%; range, - 0.2–4.3%), cancer mortality (median, 1.3%; range: - 0.3–3.9%), and respiratory disease mortality (median, 1.2%; range: - 0.3–6.1%). The proportion mediated of the total effect of vitamin D insufficiency on the mortality outcomes was generally lower than for vitamin D deficiency. Across all biomarkers of SIR and mortality outcomes, it ranged from - 3.3 to 3.6%, with a median of almost 0 (- 0.25%).

## Discussion

### Summary of the findings

With data from almost 400,000 individuals from the UK Biobank, this study showed strong cross-sectional associations of vitamin D deficiency with disadvantageous levels of all blood cell count-based biomarkers of SIR but not with the CRP-based biomarkers. With the exception of the SII, no biomarker of SIR was associated with vitamin D insufficiency.

Vitamin D deficiency, vitamin D insufficiency, and disadvantageous levels of all biomarkers of SIR were strongly associated with increased all-cause mortality, CVD, cancer, and respiratory disease mortality. After adjusting for each other, neither the association of vitamin D status with mortality nor the association of biomarkers of SIR with mortality were attenuated. In support of this finding, mediation analysis showed that the proportions of the total effects of vitamin D deficiency and insufficiency on all mortality outcomes mediated through biomarkers of SIR were close to 0% for most of the associations tested. The largest mediation proportion observed for all-cause mortality was 3.7% by the SII. This speaks against the hypothesis that biomarkers of SIR are on the pathway between vitamin D status and mortality outcomes.

### Vitamin D status and CRP-based biomarkers of SIR

Our results from the main model with adjustment of BMI and waist circumference showed that vitamin D deficiency was not associated with CRP-based biomarkers of SIR. In contrast, a cross-sectional association has been frequently observed in other observational studies. The England Longitudinal of Ageing (ELSA) study reported an association of vitamin D deficiency with elevated levels of CRP (≥ 3 mg/L) [49]. Cohort studies with hospital patients also observed an inverse association between 25(OH)D and CRP levels [72, 73]. Moreover, a Mendelian randomization study with the UK Biobank population showed that genetically predicted serum 25(OH)D levels ≤ 25 nmol/L were inversely associated with serum CRP levels [50]. However, findings from meta-analyses of RCTs speak against a causal association between vitamin D supplementation and CRP in the general population. A meta-analysis of 24 RCTs did not find such an association [74]. However, if meta-analyses of RCTs are restricted to populations with specific diseases, such as diabetes, abnormal glucose homeostasis, and psychiatric disorders, statistically significant inverse associations between vitamin D supplementation and CRP were observed [75–77].

Taken together, this speaks for a causal association of vitamin D and CRP in specific, diseased populations, in which CRP levels are increased due to the diseases. However, this does not apply to general population cohorts like the UK Biobank, in which the association of vitamin D deficiency and CRP is confounded by body weight. One reason why the Mendelian randomization study in the UK Biobank observed an association [50], and we did not, may be as follows: the authors only observed an association of genetically predicted serum 25(OH)D levels and CRP in subjects with 25(OH)D levels ≤ 25 nmol/L but not at higher 25(OH)D levels. Subjects with 25(OH)D levels ≤ 25 nmol/L likely have a high disease burden because such low 25(OH)D levels are usually observed among patients with diseases.

### Vitamin D status and blood cell count-based biomarkers of SIR

To our knowledge, our study is the first population-based cohort reporting that vitamin D deficiency is cross-sectionally associated with blood cell count-based biomarkers of SIR. We can only compare our results to previous observational studies with diseased populations, which investigated NLR and PLR. Akbas et al. showed that PLR and NLR are increased in subjects with vitamin D insufficiency in 4120 hospitalized patients [48]. Furthermore, a low vitamin D status was associated with higher NLR in patients with prediabetes/diabetes, and patients admitted to intensive care units with SARS-CoV-2 Infection [78, 79]. Furthermore, there has been a first placebo-controlled trial including 106 patients hospitalized with COVID-19 that showed vitamin D supplements decreased NLR within 2 months [80].

### Can the association of vitamin D deficiency and mortality be explained by a systemic inflammatory response to adverse health conditions?

We observed a cross-sectional association of vitamin D deficiency with disadvantageous levels of blood cell count-based biomarkers of SIR. In theory, such an association could be due to different reasons, such as (1) a disease could have caused both, inflammation and vitamin D deficiency, (2) vitamin D deficiency could have caused the inflammation, and (3) the inflammation could have caused the vitamin D deficiency. Unfortunately, no causal interferences are possible with our observational study and the question, which, if any, of these explanations might apply cannot be answered with certainty based on our results.

Nevertheless, we can approach the research question, of whether the associations of vitamin D and biomarkers of SIR with mortality are independent, with our study design. By putting them in the same Cox regression model, no attenuations of the HRs with mortality of neither biomarkers of SIR nor vitamin D status were observed. This finding was further supported by the mediation analysis, which observed very low proportions of the total effects of vitamin D deficiency and insufficiency on all mortality outcomes mediated through biomarkers of SIR. Taken together, our study does not support the hypothesis that biomarkers of SIR are on the pathway from vitamin D deficiency to mortality in the general population. However, this might be different in patient populations with high inflammation, such as individuals with cancer, diabetes mellitus, or acute cardiovascular disease [24, 26–30]. Such disease-specific cohort studies are still needed to confirm our findings.

### Strengths and limitations

This study has strengths and limitations. This is the largest cohort study with the most comprehensive list of biomarkers of SIR to date to examine the association between vitamin D status and biomarkers of SIR. The consistent findings for CRP-based and blood cell count-based biomarkers of SIR, as well as the correction for multiple testing limit the risk of chance findings for a single biomarker. Additional strengthes of the study are the availability of the long-term mortality follow-up (> 10 years) and the adjustment for 51 potential confounders in vitamin D analyses, including rarely assessed factors such as time spent outdoors in summer.

This study also has limitations. A well-known one is a healthy volunteer selection bias in the UK Biobank’s baseline study population. Although this may strongly affect absolute effect estimates (such as the prevalence of vitamin D deficiency, which is likely underestimated) the potential impact on relative effect estimates like ORs and HRs would be expected to be much smaller.

## Conclusions

This large cohort study observed cross-sectional associations of vitamin D deficiency with disadvantageous levels of blood cell count-based biomarkers of SIR. Furthermore, the strong associations of low vitamin D status with all-cause and cause-specific mortality were not attenuated when biomarkers of SIR were added to the model, and vice versa. In causal mediation analysis, the proportions of total effects of vitamin D deficiency and insufficiency on the mortality outcomes mediated by biomarkers of SIR were mostly close to 0%. Taken together, our study suggests that low vitamin D status and disadvantageous levels of biomarkers of SIR are independently associated with all-cause and cause-specific mortality. Future studies should thoroughly evaluate these associations in a cohort of patients with specific diseases that can cause a SIR (e.g., cancer).

For clinical practice, the potential of clinical interventions against both vitamin D deficiency and the underlying causes of systemic inflammation in people with both conditions should be explored.

## Supplementary Information

Below is the link to the electronic supplementary material.

Supplementary file1 (DOCX 77 kb)
