# Supplementary material for: About the associations of vitamin D deficiency and biomarkers of systemic inflammatory response with all-cause and cause-specific mortality in a general population sample of almost 400,000 UK Biobank participants
Source: Eur J Epidemiol. 2023 Jun 21;38(9):957–71. doi: 10.1007/s10654-023-01023-2 (PMC10501954; doi:10.1007/s10654-023-01023-2)
Supplement: Supplementary file 1 — Supplementary file1 (DOCX 77 kb) [file 10654_2023_1023_MOESM1_ESM.docx]

###### Supplemental Material

Table of contents

[Suppl. Table 1. Complete list of baseline characteristics of the study population 2](#_Toc137733707)

[Suppl. Table 2. Associations of dichotomized biomarkers of systemic inflammatory response with all-cause and cause-specific mortality, by age group 7](#_Toc137733708)

[Suppl. Table 3. Associations of dichotomized biomarkers of systemic inflammatory response with all-cause and cause-specific mortality, by sex 8](#_Toc137733709)

[Suppl. Table 4. Associations of vitamin D deficiency in comparison to sufficient vitamin D status with biomarkers of systemic inflammatory response in logistic regression models, stratifying by age, N_total_=261,045 9](#_Toc137733710)

[Suppl. Table 5. Associations of vitamin D deficiency in comparison to sufficient vitamin D status with biomarkers of systemic inflammatory response in logistic regression models, stratifying by sex, N_total_=261,045 10](#_Toc137733711)

[Suppl. Table 6. Estimates of mediation of biomarkers of systemic inflammatory response in the association between vitamin D deficiency (versus sufficient vitamin D status) with all-cause mortality and cause-specific mortality 11](#_Toc137733712)

[Suppl. Table 7. Estimates of mediation of biomarkers of systemic inflammatory response in the association between vitamin D insufficiency (versus sufficient vitamin D status) with all-cause mortality and cause-specific mortality 13](#_Toc137733713)

## **Suppl. Table 1. Complete list of baseline characteristics of the study population**

| **Variables** | **Study population**  **(N=397,737)** |  |
| --- | --- | --- |
|  | **N (%) / Median (IQR)** |  |
| **SOCIO-DEMOGRAPHIC/-ECONOMIC FACTORS** | |  |
| **Age (years), median (IQR)** | 58 (50, 63) |  |
| **Sex, n (%)** |  |  |
| Female | 210,982 (53.1) |  |
| Male | 186,755 (47.0) |  |
| **Education (years), median (IQR)** | 3 (1, 5) |  |
| **Townsend deprivation index (points), median (IQR)** | -2.2 (-3.7, 0.5) |  |
| **No. of individuals in household, n (%)** |  |  |
| 1 | 72,736 (18.4) |  |
| 2 | 184,281 (46.7) |  |
| 3-4 | 116,217 (29.5) |  |
| ≥ 5 | 21,312(5.4) |  |
| **Annual household income (***£***), n (%)** |  |  |
| < 18,000 | 76,625 (22.5) |  |
| 18,000 - < 30,999 | 86,043 (25.3) |  |
| 31,000- < 51,999 | 89,022 (26.2) |  |
| 52,000 - < 100,000 | 69,973 (20.6) |  |
| ≥ 100,000 | 18,668 (5.5) |  |
|  |  |  |
| **LIFE-STYLE FACTORS** |  |  |
| **Smoking, n (%)** |  |  |
| Never | 217,643 (54.8) |  |
| Occasionally | 56,401 (14.2) |  |
| Regularly | 123,091 (31.0) |  |
| **Alcohol consumption (g ethanol/d), n (%)** |  |  |
| Abstainer | 122,438 (30.9) |  |
| Women 0 - < 20 / men 0 - < 40 | 159,230 (40.1) |  |
| Women 20 - < 40 / men 40 - < 60 | 67,419 (17.0) |  |
| Women ≥ 40 / men ≥ 60 | 47,679 (12.0) |  |
| **Total physical activity (hours/day), n (%)** |  |  |
| ≤ 1 | 60,491 (18.8) |  |
| ≤ 2 | 130,989 (40.7) |  |
| > 2 | 130,419 (40.5) |  |
| **Frequency of visiting friends/family, n (%)** |  |  |
| Almost daily | 33,213 (8.4) |  |
| 2-4 times/week | 53,314 (13.5) |  |
| Once/week | 140,953 (35.7) |  |
| Once every few months/rare | 167373 (42.4) |  |
| **Oily fish consumption, n (%)** |  |  |
| Never/ less than once a week | 174,21 (44.1) |  |
| At least once a week | 220,731 (55.9) |  |
| **Cereal consumption (bowls/week), n (%)** |  |  |
| Never | 67,68 (17.1) |  |
| < 7 | 176,728 (44.7) |  |
| ≥ 7 | 151,373 (38.3) |  |
| **Processed meat intake, n (%)** |  |  |
| Never/ less than once a week | 156,518 (39.5) |  |
| At least once a week | 239,834 (60.5) |  |
| **Milk consumption, n (%)** |  |  |
| Never/rarely | 12,96 (3.3) |  |
| Occasionally/regularly | 383,977 (96.7) |  |
| **Spread consumption, n (%)** |  |  |
| Never/rarely | 42,863 (10.8) |  |
| Butter | 143,089 (36.1) |  |
| Margarine/others | 210,525 (53.1) |  |
| **Preferred bread type, n (%)** |  |  |
| White | 101,670 (26.5) |  |
| Wholemeal/wholegrain/brown | 281,481 (73.5) |  |
|  |  |  |
| **DISEASES & DISEASE SYMPTOMS** |  |  |
| **Diabetes, n (%)** |  |  |
| No | 377,677 (95.0) |  |
| Yes | 19,953 (5.0) |  |
| **Cancer, n (%)** |  |  |
| No | 366,221 (92.5) |  |
| Yes | 29,71 (7.5) |  |
| **Stroke, n (%)** |  |  |
| No | 392,258 (98.7) |  |
| Yes | 5,372 (1.4) |  |
| **CHD, n (%)** |  |  |
| No | 378,891 (95.3) |  |
| Yes | 18,739 (4.7) |  |
| **COPD, n (%)** |  |  |
| No | 396,296 (99.7) |  |
| Yes | 1,334 (0.3) |  |
| **History of pulmonary embolism, n (%)** |  |  |
| No | 394,403 (99.2) |  |
| Yes | 3,227 (0.8) |  |
| **Inflammatory bowel disease, n (%)** |  |  |
| No | 397,488 (> 99.9) |  |
| Yes | 142 (< 0.01) |  |
| **Periodontitis, n (%)** |  |  |
| No | 338,837 (85.3) |  |
| Yes | 58,496 (14.7) |  |
| **Arthritis, n (%)** |  |  |
| No | 356,086 (89.6) |  |
| Yes | 41,544 (10.5) |  |
| **Osteoporosis, n (%)** |  |  |
| No | 396,296 (99.7) |  |
| Yes | 1,334 (0.3) |  |
| **Gout, n (%)** |  |  |
| No | 391,196 (98.4) |  |
| Yes | 6,434 (1.6) |  |
| **Parkinson, n (%)** |  |  |
| No | 396,810 (99.8) |  |
| Yes | 820 (0.2) |  |
| **Depressed mood in last 2 weeks, n (%)** |  |  |
| ≤ half the days | 360,268 (95.1) |  |
| > half the days | 18,754 (5.0) |  |
| **Tiredness/lethargy in last 2 weeks, n (%)** |  |  |
| ≤ half the days | 360,268 (95.1) |  |
| > half the days | 18,754 (5.0) |  |
|  |  |  |
| **BIOMARKERS** |  |  |
| **BMI (kg/m^2^), n (%)** |  |  |
| Underweight, < 18.5 | 2,029 (0.5) |  |
| Low normal weight, 18.5 - <20 | 7,195 (1.8) |  |
| High normal weight, 20 - < 25 | 122,032 (30.8) |  |
| Overweight/obesity class I: 25 - < 35 | 237,590 (60.0) |  |
| Obesity class II: 35 - < 40 | 19,710 (5.0) |  |
| Obesity class III: ≥ 40 | 7,640 (1.9) |  |
| **Waist circumference (cm), median (IQR)** | 89 (80, 98) |  |
| **eGFR (ml/min/1,73 m^2^), n (%)** |  |  |
| ≥ 90 | 236,348 (59.5) |  |
| < 90 | 161,126 (40.5) |  |
| **HbA_1c_, (%), n (%)** |  |  |
| < 6 | 346,935 (91.9) |  |
| 6 - < 6.5 | 15,723 (4.2) |  |
| 6.5 - < 7 | 5,549 (1.5) |  |
| 7 - < 8 | 5,412 (1.4) |  |
| ≥ 8 | 3,915 (1) |  |
| **HDL cholesterol (mg/dl), n (%)** |  |  |
| < 40 | 50,361 (12.7) |  |
| ≥ 40 | 347,088 (87.3) |  |
| **SBP (mmHg), n (%)** |  |  |
| < 140 | 210,366 (53.0) |  |
| 140 - < 160 | 125,839 (31.7) |  |
| 160 - < 180 | 48,493 (12.2) |  |
| ≥ 180 | 12,584 (3.2) |  |
| **DBP (mmHg), n (%)** |  |  |
| < 90 | 302,37 (76.1) |  |
| 90 - < 100 | 72,118 (18.2) |  |
| ≥ 100 | 22,805 (5.7) |  |
| **FEV1 (L), median (IQR)** | 2.8 (2.3, 3.3) |  |
| **Hand grip strength (Kg), median (IQR)** | 31 (24, 41) |  |
|  |  |  |
| **GENERAL HEALTH** |  |  |
| **No of drugs, median (IQR)** | 2 (0, 4) |  |
| **No of chronic diseases, median (IQR)** | 2 (1, 3) |  |
| **Disability (%)** |  |  |
| No | 370,723 (94.1) |  |
| Yes | 23,102 (5.9) |  |
| **General self-reported health, n (%)** |  |  |
| Excellent | 65,495 (16.6) |  |
| Good | 229,374 (58.0) |  |
| Fair | 83,086 (21.0) |  |
| Poor | 17,458 (4.4) |  |
| **VITAMIN D SPECIFIC FACTORS** |  |  |
| **Latitude of study center (per 1°), median (IQR)** | 53.0 (51.5, 53.8) |  |
| **Month of attending the study center** |  |  |
| 1 | 27,084 (6.8) |  |
| 2 | 31,850 (8.0) |  |
| 3 | 40,027 (10.1) |  |
| 4 | 35,422 (8.9) |  |
| 5 | 42,495 (10.7) |  |
| 6 | 41,636 (10.5) |  |
| 7 | 34,489 (8.7) |  |
| 8 | 28,381 (7.1) |  |
| 9 | 27,375 (6.9) |  |
| 10 | 33,727 (8.5) |  |
| 11 | 32,964 (8.3) |  |
| 12 | 22,287 (5.6) |  |
| **25(OH)D, nmol/L, median (IQR)** | 46.8 (32.3, 62.4) |  |
| **Vitamin D status, n (%)** |  |  |
| Vitamin D deficiency | 83,929 (21.1) |  |
| Vitamin D insufficiency | 136,692 (34.4) |  |
| Vitamin D sufficiency | 177,116 (44.5) |  |
| **Time spent outdoors in summer (h/day), n (%)** |  |  |
| <1 | 16,573 (4.4) |  |
| 1-2 | 115,708 (30.9) |  |
| 3-4 | 123,575 (33.0) |  |
| 5-6 | 75,304 (20.1) |  |
| ≥ 7 | 43,404 (11.6) |  |
| **Time spent outdoors in winter (h/day), n (%)** |  |  |
| <1 | 74,601 (19.9) |  |
| 1-2 | 212,54 (56.8) |  |
| 3-4 | 56,614 (15.1) |  |
| ≥ 5 | 30,649 (8.2) |  |
| **Skin color, n (%)** |  |  |
| Very fair | 30,256 (7.7) |  |
| Fair | 267,131 (68.2) |  |
| Olive | 72,673 (18.6) |  |
| Brown | 7,403 (1.9) |  |
| Black | 11,100 (2.8) |  |
| Unknown | 3,032 (0.8) |  |
| **Ease of skin tanning, n (%)** |  |  |
| Very tanned | 84,012 (21.7) |  |
| Moderately tanned | 154,199 (39.9) |  |
| Mildly/occasionally tanned | 81,754 (21.2) |  |
| Never tan, only burn | 66,484 (17.2) |  |
| **Sun screen/UV protection use, n (%)** |  |  |
| Never/rarely | 40,309 (10.2) |  |
| Sometimes | 132,512 (33.4) |  |
| Most of times | 139,854 (35.3) |  |
| Always | 81,439 (20.5) |  |
| Do not go out in sunshine | 2,384 (0.6) |  |
| **Solarium/sunlamp use (times per year), n (%)** |  |  |
| Never | 356,176 (90.7) |  |
| < 1 | 19,253 (4.9) |  |
| 1 - 6 | 9,534 (2.4) |  |
| 7 - 12 | 4,099 (1.0) |  |
| > 12 | 3,872 (1.0) |  |
| **BIOMARKERS OF SYSTEMIC INFLAMMATORY RESPONSE** |  |  |
| **CRP-based biomarkers** |  |  |
| CRP (mg/L) | 1.3 (0.7, 2.8) |  |
| mGPS |  |  |
| 0 | 381,157 (95.8) |  |
| 1 | 16,496 (4.2) |  |
| 2 | 84 (0.0) |  |
| HS_mGPS |  |  |
| 0 | 307,861 (77.4) |  |
| 1 | 89,728 (22.6) |  |
| 2 | 148 (0.0) |  |
| **Blood cell-based biomarkers** |  |  |
| NLR | 2.1 (1.7, 2.8) |  |
| PLR | 132.3 (105.4, 166.5) |  |
| LMR | 4.2 (3.2, 5.3) |  |
| SII | 529.0 (392.2, 716.8) |  |
| PNI | 54.7 (52.2, 57.4) |  |
| NPS |  |  |
| 0 | 382,192 (96.1) |  |
| 1 | 14,811 (3.7) |  |
| 2 | 734 (0.2) |  |

Abbreviations: 25(OH)D: 25-hydroxyvitamin D, BMI: body mass index, CHD: coronary heart disease, COPD: chronic obstructive pulmonary disease, CRP: C-reactive protein, DBP: diastolic blood pressure, eGRF: estimated Glomerular filtration rate, FEV1: Forced expiratory volume in 1-second, HS_mGPS: High-sensitive mGPS, IQR: interquartile range, LMR: lymphocyte-to-monocyte ratio, mGPS: modified Glasgow prognostic score, NLR: neutrophil-to-lymphocyte ratio, NPS: neutrophil-platelet score, PLR: platelet-to-lymphocyte ratio, PNI: prognostic nutritional index, SBP: systolic blood pressure, SII: systemic immune-inflammation index.

## **Suppl. Table 2. Associations of dichotomized biomarkers of systemic inflammatory response with all-cause and cause-specific mortality, by age group**

|  | **Biomarkers of systemic inflammatory response, HR (95%CI) ^a^** | | | | | | | | | |
| --- | --- | --- | --- | --- | --- | --- | --- | --- | --- | --- |
| **Mortality** | | **CRP**  > 2.75 mg/L | **mGPS**  ≥ 1 | **HS_mGPS**  ≥ 1 | **NLR**  > 2.78 | **PLR**  > 237 | **LMR**  < 2.56 | **SII**  > 717 | **PNI**  < 50 | **NPS**  ≥ 1 |
| *Age, 37-64 years* | |  |  |  |  |  |  |  |  |  |
| **All-cause**  **(N**_deaths_**=** **17,249)** | | 1.73 (1.67,1.79) | 1.74 (1.69,1.80) | 1.80 (1.72,1.87) | 1.52 (1.48,1.57) | 2.02 (1.91,2.13) | 1.74 (1.65,1.84) | 1.82 (1.74,1.89) | 1.44 (1.40,1.49) | 1.98 (1.87,2.09) |
| **CVD**  **(N**_deaths_**=** **3,402)** | | 1.81 (1.68,1.95) | 1.80 (1.67,1.94) | 1.97 (1.80,2.14) | 1.79 (1.67,1.92) | 2.20 (1.95,2.49) | 1.74 (1.52,1.98) | 1.87 (1.71,2.06) | 1.65 (1.54,1.77) | 1.87 (1.65,2.11) |
| **Cancer**  **(N**_deaths_**= 9,185)** | | 1.65 (1.57,1.73) | 1.65 (1.57,1.72) | 1.57 (1.48,1.67) | 1.35 (1.29,1.41) | 1.66 (1.53,1.81) | 1.63 (1.51,1.77) | 1.57 (1.48,1.67) | 1.31 (1.25,1.37) | 1.79 (1.65,1.94) |
| **Respiratory**  **(N**_deaths_**= 1,062)** | | 2.95 (2.59,3.36) | 2.99 (2.62,3.41) | 2.50 (2.16,2.89) | 2.15 (1.91,2.43) | 4.33 (3.67,5.10) | 2.37 (1.95,2.89) | 2.33 (2.00,2.73) | 2.28 (2.02,2.57) | 3.39 (2.82,4.07) |
|  | |  |  |  |  |  |  |  |  |  |
| *Age, 65-73 years* | |  |  |  |  |  |  |  |  |  |
| **All-cause**  **(N**_deaths_**=** **12,299)** | | 1.44 (1.38,1.49) | 1.44 (1.39,1.50) | 1.44 (1.38,1.51) | 1.42 (1.37,1.47) | 1.91 (1.79,2.05) | 1.44 (1.34,1.54) | 1.38 (1.31,1.44) | 1.42 (1.37,1.47) | 1.82 (1.70,1.94) |
| **CVD**  **(N**_deaths_**= 2,689)** | | 1.44 (1.33,1.56) | 1.49 (1.38,1.62) | 1.61 (1.47,1.77) | 1.59 (1.47,1.72) | 2.19 (1.90,2.51) | 1.26 (1.07,1.48) | 1.38 (1.25,1.52) | 1.53 (1.41,1.65) | 1.71 (1.49,1.96) |
| **Cancer**  **(N**_deaths_**= 5,710)** | | 1.43 (1.35,1.51) | 1.43 (1.35,1.52) | 1.33 (1.24,1.42) | 1.24 (1.17,1.31) | 1.52 (1.36,1.71) | 1.43 (1.30,1.59) | 1.33 (1.24,1.43) | 1.29 (1.22,1.37) | 1.73 (1.57,1.90) |
| **Respiratory**  **(N**_deaths_**= 1,024)** | | 2.41 (2.12,2.73) | 2.37 (2.08,2.69) | 1.83 (1.58,2.11) | 1.80 (1.59,2.04) | 3.67 (3.05,4.42) | 1.94 (1.57,2.39) | 1.59 (1.37,1.85) | 1.99 (1.76,2.26) | 3.27 (2.74,3.91) |
|  | |  |  |  |  |  |  |  |  |  |
| *Interaction* *p-value^b^* | |  |  |  |  |  |  |  |  |  |
| **All-cause** | | **<0.01** | **<0.01** | **<0.01** | 0.10 | **<0.01** | **<0.01** | 0.20 | **<0.01** | **<0.01** |
| **CVD** | | **<0.01** | 0.13 | **<0.01** | 0.70 | 0.07 | 0.32 | 0.63 | 0.09 | 0.57 |
| **Cancer** | | **<0.01** | 0.14 | **0.02** | 0.11 | 0.09 | 0.11 | 0.27 | **<0.01** | **0.04** |
| **Respiratory** | | **<0.01** | **0.04** | **<0.01** | **0.04** | 0.44 | **0.047** | 0.06 | **<0.01** | **0.01** |

Abbreviations: CI: confidence interval; CRP: C-reactive protein; CVD: cardiovascular disease; HR: hazard ratio; HS_mGPS: High-sensitive mGPS; LMR: lymphocyte-to-monocyte ratio; mGPS: modified Glasgow prognostic score; NLR: neutrophil-to-lymphocyte ratio; NPS: neutrophil-platelet score; PLR: platelet-to-lymphocyte ratio; PNI: prognostic nutritional index; SII: systemic immune-inflammation index.

1. The models are adjusted for sex, body mass index, waist circumference, and vitamin D status.
2. Statistically significant interactions with age (p<0.05) were printed in bold.

## **Suppl. Table 3. Associations of dichotomized biomarkers of systemic inflammatory response with all-cause and cause-specific mortality, by sex**

|  | **Biomarkers of systemic inflammatory response, HR (95%CI) ^a^** | | | | | | | | | |
| --- | --- | --- | --- | --- | --- | --- | --- | --- | --- | --- |
| **Mortality** | | **CRP**  > 2.75 mg/L | **mGPS**  ≥ 1 | **HS_mGPS**  ≥ 1 | **NLR**  > 2.78 | **PLR**  > 237 | **LMR**  < 2.56 | **SII**  > 717 | **PNI**  < 50 | **NPS**  ≥ 1 |
| *Females* | |  |  |  |  |  |  |  |  |  |
| **All-cause**  **(N**_deaths_**=11,648)** | | 1.42 (1.36,1.47) | 1.78 (1.66,1.89) | 1.42 (1.36,1.48) | 1.49 (1.43,1.55) | 1.67 (1.57,1.79) | 1.76 (1.65,1.87) | 1.42 (1.36,1.47) | 1.52 (1.44,1.59) | 1.89 (1.77,2.03) |
| **CVD**  **(N**_deaths_**=1,783)** | | 1.34 (1.21,1.49) | 1.58 (1.35,1.86) | 1.35 (1.22,1.50) | 1.71 (1.55,1.90) | 1.50 (1.26,1.80) | 1.97 (1.70,2.29) | 1.57 (1.43,1.74) | 1.50 (1.32,1.71) | 2.04 (1.73,2.41) |
| **Cancer**  **(N**_deaths_**=6,657)** | | 1.39 (1.32,1.47) | 1.59 (1.45,1.74) | 1.40 (1.32,1.48) | 1.31 (1.24,1.38) | 1.61 (1.47,1.76) | 1.56 (1.43,1.70) | 1.26 (1.20,1.33) | 1.40 (1.31,1.50) | 1.55 (1.40,1.71) |
| **Respiratory (N**_deaths_**=730)** | | 2.60 (2.21,3.05) | 3.59 (2.93,4.40) | 2.50 (2.13,2.94) | 2.16 (1.85,2.51) | 2.27 (1.80,2.86) | 2.45 (1.99,3.03) | 2.18 (1.89,2.53) | 1.81 (1.50,2.18) | 3.99 (3.26,4.89) |
|  | |  |  |  |  |  |  |  |  |  |
| *Males* | |  |  |  |  |  |  |  |  |  |
| **All-cause**  **(N**_deaths_**=17,900)** | | 1.66 (1.61,1.71) | 1.98 (1.88,2.09) | 1.67 (1.62,1.73) | 1.44 (1.39,1.48) | 1.55 (1.47,1.65) | 1.46 (1.41,1.51) | 1.46 (1.42,1.51) | 1.49 (1.43,1.55) | 2.14 (2.02,2.26) |
| **CVD**  **(N**_deaths_**=4,308)** | | 1.72 (1.62,1.84) | 1.91 (1.72,2.14) | 1.75 (1.64,1.87) | 1.64 (1.54,1.74) | 1.50 (1.33,1.69) | 1.62 (1.51,1.73) | 1.61 (1.51,1.71) | 1.51 (1.39,1.63) | 2.35 (2.11,2.62) |
| **Cancer**  **(N**_deaths_**=8,238)** | | 1.62 (1.54,1.70) | 1.88 (1.73,2.04) | 1.62 (1.54,1.70) | 1.27 (1.22,1.33) | 1.50 (1.38,1.64) | 1.30 (1.24,1.37) | 1.36 (1.30,1.42) | 1.35 (1.27,1.43) | 1.77 (1.62,1.94) |
| **Respiratory (N**_deaths_**=1,356)** | | 2.56 (2.29,2.86) | 3.05 (2.58,3.59) | 2.60 (2.32,2.90) | 1.82 (1.64,2.03) | 2.03 (1.69,2.44) | 1.86 (1.65,2.09) | 2.10 (1.89,2.34) | 1.72 (1.50,1.97) | 4.14 (3.55,4.84) |
|  | |  |  |  |  |  |  |  |  |  |
| *Interaction* *p-value* | |  |  |  |  |  |  |  |  |  |
| **All-cause** | | **<0.01** | **<0.01** | **<0.01** | 0.38 | 0.18 | **<0.01** | 0.10 | 0.87 | **<0.01** |
| **CVD** | | **<0.01** | **0.03** | **<0.01** | 0.41 | 0.96 | **<0.01** | 0.69 | 0.89 | 0.12 |
| **Cancer** | | **<0.01** | **<0.01** | **<0.01** | 0.84 | 0.36 | **<0.01** | **0.02** | 0.94 | **0.03** |
| **Respiratory** | | 0.46 | 0.12 | 0.87 | 0.11 | 0.79 | **0.04** | 0.75 | 0.77 | 0.74 |

Abbreviations: CI: confidence interval; CRP: C-reactive protein; CVD: cardiovascular disease; HR: hazard ratio; HS_mGPS: High-sensitive mGPS; LMR: lymphocyte-to-monocyte ratio; mGPS: modified Glasgow prognostic score; NLR: neutrophil-to-lymphocyte ratio; NPS: neutrophil-platelet score; PLR: platelet-to-lymphocyte ratio; PNI: prognostic nutritional index; SII: systemic immune-inflammation index.

1. The models are adjusted for age, body mass index, waist circumference, and vitamin D status.
2. Statistically significant interactions with sex (p<0.05) were printed in bold.

## **Suppl. Table 4. Associations of vitamin D deficiency in comparison to sufficient vitamin D status with biomarkers of systemic inflammatory response in logistic regression models, stratifying by age, N**_total_**=261,045**

|  | | **Vitamin D deficiency, N=83,929** | | |  |
| --- | --- | --- | --- | --- | --- |
| **Biomarkers of systemic  inflammatory response** | | **Age, 37-64 years**  N= 71,494 | **Age, 65-73 years**  N= 12,435 |  |  |
| ***N****_total_* |  | **OR (95%CI)** ^b^**, FDR** | **OR (95%CI)** ^b^**, FDR** | **Interaction  FDR** ^c^ |  |
| **CRP based** | **CRP** | 1.02 (1.00, 1.05), 0.148 | 0.98 (0.93, 1.03), 0.528 | 0.008 |  |
|  | **mGPS** | 0.99 (0.94, 1.05), 0.882 | 0.97 (0.87, 1.07), 0.584 | 0.009 |  |
|  | **HS_mGPS** | 1.03 (1.00, 1.06), 0.068 | 1.00 (0.95, 1.06), 0.882 | 0.125 |  |
| **Blood cell** | **NPS** | 1.12 (1.05, 1.18), <.001 | 1.16 (1.04, 1.30), 0.021 | 0.205 |  |
|  | **NLR** | 1.10 (1.07, 1.13), <.001 | 1.10 (1.05, 1.16), <.001 | 0.180 |  |
|  | **PLR** | 1.09 (1.04, 1.15), 0.002 | 1.10 (0.99, 1.22), 0.133 | 0.237 |  |
|  | **LMR** | 1.05 (1.01, 1.09), 0.036 | 1.00 (0.93, 1.07), 0.996 | 0.483 |  |
|  | **SII** | 1.16 (1.13, 1.19), <.001 | 1.16 (1.10, 1.23), <.001 | 0.676 |  |
|  | **PNI** | 1.09 (1.04, 1.13), <.001 | 1.03 (0.96, 1.11), 0.483 | 0.017 |  |

Abbreviations: CRP: C-reactive protein, LMR: lymphocyte-to-monocyte ratio, mGPS: modified Glasgow prognostic score, NLR: neutrophil-to-lymphocyte ratio, NPS: neutrophil-platelet score, OR: odds ratio, PLR: platelet-to-lymphocyte ratio, PNI: prognostic nutritional index, SII: systemic immune-inflammation index.

^a^ N=136,692 participants with vitamin D sufficiency (reference group) not shown.

^b^ The model is adjusted for covariates in Model 4 (see legend of Table 4)

^c^ Age was considered as continuous variable when calculating interaction terms.

## **Suppl. Table 5. Associations of vitamin D deficiency in comparison to sufficient vitamin D status with biomarkers of systemic inflammatory response in logistic regression models, stratifying by sex, N**_total_**=261,045**

|  | | **Vitamin D deficiency, N=83,929 ^a^** | | |  |
| --- | --- | --- | --- | --- | --- |
| **Biomarkers of systemic  inflammatory response** | | **Females**  N=44,195 | **Males**  N=39,734 |  |  |
| ***N****_total_* |  | **OR (95%CI)** ^b^**, FDR** | **OR (95%CI)** ^b^**, FDR** | **Interaction  FDR** |  |
| **CRP based** | **CRP** | 0.97 (0.94, 1.01), 0.175 | 1.03 (0.99, 1.07), 0.148 | 0.216 |  |
|  | **mGPS** | 0.95 (0.89, 1.01), 0.152 | 1.00 (0.92, 1.08), 0.987 | 0.036 |  |
|  | **HS_mGPS** | 0.98 (0.95, 1.01), 0.318 | 1.05 (1.01, 1.09), 0.039 | 0.308 |  |
| **Blood cell** | **NPS** | 1.10 (1.03, 1.17), 0.015 | 1.13 (1.04, 1.22), 0.010 | 0.469 |  |
|  | **NLR** | 1.12 (1.08, 1.16), <.001 | 1.08 (1.04, 1.11), <.001 | <.001 |  |
|  | **PLR** | 1.14 (1.07, 1.21), <.001 | 1.03 (0.96, 1.11), 0.483 | <.001 |  |
|  | **LMR** | 1.11 (1.04, 1.17), 0.003 | 1.01 (0.97, 1.05), 0.744 | <.001 |  |
|  | **SII** | 1.19 (1.15, 1.23), <.001 | 1.11 (1.07, 1.15), <.001 | <.001 |  |
|  | **PNI** | 1.08 (1.03, 1.13), 0.004 | 1.07 (1.01, 1.12), 0.035 | <.001 |  |

Abbreviations: CRP: C-reactive protein, LMR: lymphocyte-to-monocyte ratio, mGPS: modified Glasgow prognostic score, NLR: neutrophil-to-lymphocyte ratio, NPS: neutrophil-platelet score, OR: odds ratio, PLR: platelet-to-lymphocyte ratio, PNI: prognostic nutritional index, SII: systemic immune-inflammation index.

^a^ N=136,692 participants with vitamin D sufficiency (reference group) not shown.

^b^ The model is adjusted for covariates in Model 4 (see legend of Table 4)

## **Suppl. Table 6. Estimates of mediation of biomarkers of systemic inflammatory response in the association between vitamin D deficiency (versus sufficient vitamin D status) with all-cause mortality and cause-specific mortality**

| **Mortality outcomes** | **Mediator** | **Natural direct  effect ^a^** | **Natural indirect  effect ^b^** | **Total  effect ^c^** | **Proportion mediated ^d^** |
| --- | --- | --- | --- | --- | --- |
|  |  | **HR (95%CI) ^e^** | **HR ^e^** | **HR (95%CI) ^e^** | **%** |
| **All-cause** | **CRP** | 1.35 (1.30,1.39) | 1.00 | 1.35 (1.30,1.40) | 0.3 |
|  | **mGPS** | 1.34 (1.30,1.39) | 1.00 | 1.34 (1.30,1.39) | -0.3 |
|  | **HS_mGPS** | 1.34 (1.30,1.39) | 1.00 | 1.35 (1.30,1.39) | 0.5 |
|  | **NPS** | 1.35 (1.30,1.39) | 1.00 | 1.35 (1.30,1.40) | 0.9 |
|  | **NLR** | 1.34 (1.29,1.38) | 1.01 | 1.34 (1.30,1.39) | 2.6 |
|  | **PLR** | 1.34 (1.30,1.39) | 1.00 | 1.35 (1.31,1.40) | 1.5 |
|  | **LMR** | 1.34 (1.29,1.38) | 1.00 | 1.34 (1.29,1.39) | 1.1 |
|  | **SII** | 1.34 (1.29,1.38) | 1.01 | 1.35 (1.30,1.40) | 3.7 |
|  | **PNI** | 1.34 (1.30,1.39) | 1.00 | 1.35 (1.30,1.39) | 1.6 |
| **CVD** | **CRP** | 1.20 (1.14,1.26) | 1.00 | 1.20 (1.14,1.26) | 0.4 |
|  | **mGPS** | 1.39 (1.29,1.50) | 1.00 | 1.39 (1.29,1.50) | -0.2 |
|  | **HS_mGPS** | 1.39 (1.29,1.50) | 1.00 | 1.39 (1.29,1.50) | 0.6 |
|  | **NPS** | 1.40 (1.30,1.51) | 1.00 | 1.41 (1.31,1.52) | 0.9 |
|  | **NLR** | 1.39 (1.29,1.50) | 1.01 | 1.40 (1.30,1.51) | 3.0 |
|  | **PLR** | 1.40 (1.30,1.51) | 1.00 | 1.41 (1.31,1.52) | 1.1 |
|  | **LMR** | 1.40 (1.30,1.51) | 1.00 | 1.40 (1.30,1.51) | 1.0 |
|  | **SII** | 1.39 (1.29,1.50) | 1.01 | 1.41 (1.31,1.52) | 4.3 |
|  | **PNI** | 1.40 (1.30,1.51) | 1.00 | 1.41 (1.30,1.51) | 1.2 |
| **Cancer** | **CRP** | 1.20 (1.14,1.26) | 1.00 | 1.20 (1.14,1.26) | 0.4 |
|  | **mGPS** | 1.20 (1.14,1.25) | 1.00 | 1.19 (1.14,1.25) | -0.3 |
|  | **HS_mGPS** | 1.20 (1.14,1.26) | 1.00 | 1.20 (1.14,1.26) | 0.7 |
|  | **NPS** | 1.19 (1.14,1.25) | 1.00 | 1.19 (1.14,1.25) | 1.0 |
|  | **NLR** | 1.19 (1.13,1.25) | 1.00 | 1.20 (1.14,1.25) | 2.3 |
|  | **PLR** | 1.19 (1.14,1.25) | 1.00 | 1.20 (1.14,1.26) | 2.5 |
|  | **LMR** | 1.19 (1.13,1.25) | 1.00 | 1.19 (1.13,1.25) | 1.3 |
|  | **SII** | 1.19 (1.13,1.25) | 1.01 | 1.20 (1.14,1.26) | 3.9 |
|  | **PNI** | 1.19 (1.13,1.25) | 1.00 | 1.19 (1.14,1.25) | 1.9 |
| **Respiratory** | **CRP** | 1.67 (1.47,1.90) | 1.00 | 1.68 (1.47,1.91) | 0.4 |
| **diseases** | **mGPS** | 1.67 (1.47,1.90) | 1.00 | 1.67 (1.47,1.90) | -0.3 |
|  | **HS_mGPS** | 1.67 (1.47,1.90) | 1.00 | 1.68 (1.47,1.91) | 0.7 |
|  | **NPS** | 1.70 (1.49,1.93) | 1.00 | 1.70 (1.50,1.93) | 1.0 |
|  | **NLR** | 1.62 (1.42,1.84) | 1.01 | 1.64 (1.44,1.86) | 3.2 |
|  | **PLR** | 1.65 (1.46,1.87) | 1.01 | 1.66 (1.47,1.88) | 1.6 |
|  | **LMR** | 1.63 (1.43,1.85) | 1.00 | 1.63 (1.44,1.85) | 1.2 |
|  | **SII** | 1.61 (1.41,1.82) | 1.02 | 1.65 (1.45,1.87) | 6.1 |
|  | **PNI** | 1.64 (1.45,1.86) | 1.01 | 1.65 (1.46,1.88) | 1.5 |

Abbreviations: CI: confidence interval, CRP: C-reactive protein, HR: hazard ratio, HS_mGPS: High-sensitive mGPS, LMR: lymphocyte-to-monocyte ratio, mGPS: modified Glasgow prognostic score, NA: not applicable, NLR: neutrophil-to-lymphocyte ratio, NPS: neutrophil-platelet score, PLR: platelet-to-lymphocyte ratio, PNI: prognostic nutritional index, SII: systemic immune-inflammation index.

1. The natural direct effect estimates the part of the total effect that does not operate through the mediator.
2. The natural indirect effect estimates the effect of an exposure on an outcome through its effect on the level of the mediator.
3. The total effect for a time-to-event model is the product of the natural indirect effect and the natural direct effect.
4. Proportion mediated = (natural direct effect * (natural indirect effect − 1)) / (natural direct effect * natural indirect effect − 1).
5. All models were adjusted for the covariates of Model 4 (see legend of Table 4). The first imputed dataset (imputation=1) was used in the analyses.

## **Suppl. Table 7. Estimates of mediation of biomarkers of systemic inflammatory response in the association between vitamin D insufficiency (versus sufficient vitamin D status) with all-cause mortality and cause-specific mortality**

| **Mortality outcome** | **Mediator** | **Natural direct  effect ^a^** | **Natural indirect  effect ^b^** | **Total  effect ^c^** | **Proportion mediated ^d^** |
| --- | --- | --- | --- | --- | --- |
|  |  | **HR (95%CI) ^e^** | **HR (95%CI) ^e^** | **HR (95%CI) ^e^** | **%** |
| **All-cause** | **CRP** | 1.10 (1.07,1.13) | 1.00 | 1.10 (1.07,1.13) | -1.6 |
|  | **mGPS** | 1.10 (1.07,1.13) | 1.00 | 1.10 (1.07,1.13) | -2.0 |
|  | **HS_mGPS** | 1.10 (1.07,1.13) | 1.00 | 1.10 (1.07,1.13) | -1.6 |
|  | **NPS** | 1.09 (1.06,1.13) | 1.00 | 1.09 (1.06,1.12) | -0.2 |
|  | **NLR** | 1.09 (1.06,1.12) | 1.00 | 1.09 (1.06,1.12) | 0.8 |
|  | **PLR** | 1.09 (1.06,1.13) | 1.00 | 1.09 (1.06,1.13) | -0.1 |
|  | **LMR** | 1.10 (1.07,1.13) | 1.00 | 1.10 (1.07,1.13) | -0.3 |
|  | **SII** | 1.09 (1.06,1.12) | 1.00 | 1.09 (1.06,1.12) | 2.7 |
|  | **PNI** | 1.10 (1.07,1.13) | 1.00 | 1.10 (1.06,1.13) | -0.4 |
| **CVD** | **CRP** | 1.13 (1.06,1.21) | 1.00 | 1.13 (1.06,1.21) | -1.1 |
|  | **mGPS** | 1.13 (1.06,1.20) | 1.00 | 1.13 (1.06,1.20) | -1.3 |
|  | **HS_mGPS** | 1.13 (1.06,1.21) | 1.00 | 1.13 (1.06,1.20) | -1.2 |
|  | **NPS** | 1.12 (1.05,1.19) | 1.00 | 1.12 (1.05,1.19) | -0.2 |
|  | **NLR** | 1.12 (1.05,1.19) | 1.00 | 1.12 (1.05,1.19) | 0.9 |
|  | **PLR** | 1.12 (1.05,1.20) | 1.00 | 1.12 (1.05,1.19) | -0.1 |
|  | **LMR** | 1.13 (1.06,1.20) | 1.00 | 1.13 (1.06,1.20) | -0.3 |
|  | **SII** | 1.12 (1.05,1.19) | 1.00 | 1.12 (1.05,1.19) | 3.0 |
|  | **PNI** | 1.12 (1.05,1.19) | 1.00 | 1.12 (1.05,1.19) | -0.4 |
| **Cancer** | **CRP** | 1.05 (1.01,1.09) | 1.00 | 1.05 (1.01,1.09) | -3.1 |
|  | **mGPS** | 1.05 (1.01,1.10) | 1.00 | 1.05 (1.01,1.09) | -3.3 |
|  | **HS_mGPS** | 1.05 (1.01,1.10) | 1.00 | 1.05 (1.01,1.09) | -3.0 |
|  | **NPS** | 1.05 (1.01,1.09) | 1.00 | 1.05 (1.01,1.09) | -0.2 |
|  | **NLR** | 1.05 (1.01,1.09) | 1.00 | 1.05 (1.01,1.09) | 1.1 |
|  | **PLR** | 1.05 (1.01,1.09) | 1.00 | 1.05 (1.01,1.09) | -0.2 |
|  | **LMR** | 1.05 (1.01,1.09) | 1.00 | 1.05 (1.01,1.09) | -0.3 |
|  | **SII** | 1.05 (1.01,1.09) | 1.00 | 1.05 (1.01,1.09) | 3.6 |
|  | **PNI** | 1.05 (1.01,1.09) | 1.00 | 1.05 (1.01,1.09) | -0.6 |
| **Respiratory** | **CRP** | 1.25 (1.12,1.40) | 1.00 | 1.25 (1.12,1.39) | -2.0 |
| **diseases** | **mGPS** | 1.28 (1.14,1.42) | 1.00 | 1.27 (1.14,1.42) | -2.0 |
|  | **HS_mGPS** | 1.26 (1.13,1.41) | 1.00 | 1.26 (1.13,1.41) | -1.7 |
|  | **NPS** | 1.26 (1.13,1.41) | 1.00 | 1.26 (1.13,1.40) | -0.2 |
|  | **NLR** | 1.27 (1.14,1.41) | 1.00 | 1.27 (1.14,1.42) | 0.5 |
|  | **PLR** | 1.29 (1.15,1.44) | 1.00 | 1.29 (1.15,1.44) | -0.2 |
|  | **LMR** | 1.26 (1.13,1.41) | 1.00 | 1.26 (1.13,1.41) | -0.1 |
|  | **SII** | 1.26 (1.13,1.40) | 1.01 | 1.26 (1.13,1.41) | 2.5 |
|  | **PNI** | 1.27 (1.14,1.42) | 1.00 | 1.27 (1.14,1.42) | -0.2 |

Abbreviations: CI: confidence interval, CRP: C-reactive protein, HR: hazard ratio, HS_mGPS: High-sensitive mGPS, LMR: lymphocyte-to-monocyte ratio, mGPS: modified Glasgow prognostic score, NA: not applicable, NLR: neutrophil-to-lymphocyte ratio, NPS: neutrophil-platelet score, PLR: platelet-to-lymphocyte ratio, PNI: prognostic nutritional index, SII: systemic immune-inflammation index.

1. The natural direct effect estimates the part of the total effect that does not operate through the mediator.
2. The natural indirect effect estimates the effect of an exposure on an outcome through its effect on the level of the mediator.
3. The total effect for a time-to-event model is the product of the natural indirect effect and the natural direct effect.
4. Proportion mediated = (natural direct effect * (natural indirect effect − 1)) / (natural direct effect * natural indirect effect − 1).
5. All models were adjusted for the covariates of Model 4 (see legend of Table 4). The first imputed dataset (imputation=1) was used in the analyses.
